# Efficacy of a spot-on combination of fluralaner plus moxidectin (Bravecto^®^ Plus) in cats following repeated experimental challenge with a field isolate of *Ctenocephalides felis*

**DOI:** 10.1186/s13071-019-3512-x

**Published:** 2019-05-23

**Authors:** Petr Fisara, Frank Guerino, Fangshi Sun

**Affiliations:** 1 MSD Animal Health Australia Ltd., 26 Artisan Road, Seven Hills, NSW 2147 Australia; 2 Merck Animal Health, 2 Giralda Farms, Madison, NJ 07940 USA

**Keywords:** Bravecto, Cat, *Ctenocephalides felis*, Efficacy, Fipronil, Fleas, Fluralaner, Moxidectin, *S*-methoprene

## Abstract

**Background:**

A spot-on formulation of fluralaner plus moxidectin has been designed to provide long-term protection against fleas and ticks, prevent heartworm disease and treat gastrointestinal nematode infections in cats. The objective of this study was to determine the efficacy of this product against fleas collected from a household with repeated fipronil failures following owner-administered treatments.

**Methods:**

Thirty cats were randomized to three equal groups: (A) untreated controls; (B) to receive a single application of fluralaner plus moxidectin (Bravecto^®^ Plus) at 40 mg/kg and 2 mg/kg body weight, respectively; and (C) three applications at one month intervals with a spot-on formulation of fipronil and (*S*)-methoprene (Frontline^®^ Plus) at 0.5 ml manufacturer recommended dose. Flea challenges were completed on Days −6 (for randomization), −1, 7, 14, 28, 42, 56, 70, 77, 84 and 91. Flea counts were completed 48 hours after initial treatment and 48 hours following each subsequent challenge.

**Results:**

Fleas were found on all control and all fipronil and (*S*)-methoprene treated cats at every assessment. From Day 2 to Day 93, all cats in the fluralaner plus moxidectin group were flea-free, with one exception (Day 58; three fleas counted on one cat); control group flea counts ranged between 34–109, and fipronil and (*S*)-methoprene group counts ranged between 1–79. At each assessment after Day 0, compared to the control group, geometric mean flea counts were significantly lower in the fipronil and (*S*)-methoprene group (*P* ≤ 0.04) and in the fluralaner plus moxidectin group (*P* < 0.001), and mean flea counts in the fluralaner plus moxidectin group were significantly lower than those of the fipronil and (*S*)-methoprene group (*P* < 0.001). The efficacy of fluralaner plus moxidectin, based on geometric means, was 100% at each assessment post-Day 0 except on Day 58 when efficacy was 99.7%. In the fipronil and (*S*)-methoprene group efficacy ranged between 30.6–65.6%.

**Conclusions:**

These findings demonstrate complete efficacy of fluralaner plus moxidectin against a flea isolate that was not controlled by fipronil and (*S*)-methoprene. This study provides confirmation of the consistent, sustained efficacy of topically applied fluralaner in the treatment and control of flea infestations in cats.

## Background

Exponential growth in the use of low-volume, monthly-applied, topical flea control products for cats followed the release of spot-on formulations of imidacloprid and fipronil in the mid-1990s. These compounds distribute from their application sites across the surface of the skin and kill fleas by contact and/or ingestion. The success of these and other topical spot-on products reflect a positive response from owners to the convenience of this route of parasiticide delivery in cats. The combination of fipronil with the insect growth regulator (IGR) (*S*)-methoprene, launched in the early 2000s as a spot-on, went on to become a leading product for flea control in cats. The IGR was added to maintain efficacy and reduce selection for fleas resistant to fipronil by interfering with the development of eggs laid by any adult female fleas that might have survived the effect of fipronil [1]. Nonetheless, an early report of fipronil failure and subsequent reports from clinical field studies indicate that there has been an overall decline in efficacy, regardless of whether fipronil was used alone or in combination with (*S*)-methoprene [2–7]. Potential causes of a decline in efficacy include owner failure to reliably comply with treatment instructions, the effects of climate (rain, sun) leading to more rapid degradation of the applied product, and flea infestations from external sources [3, 8]. Nonetheless, the number of publications reporting apparent reductions in the efficacy of fipronil has continued to grow, including in regions in which fipronil had previously been shown to be effective, and in cases where treatment application and climatic factors had been largely eliminated as contributory to those failures [9, 10].

The recent availability of the isoxazolines, a family of systemically-acting ectoparasiticides, therefore appears timely in light of the reports of reduced field efficacy of fipronil. The isoxazolines have been shown to paralyse and kill arthropods by blocking γ-aminobutyric acid (GABA)-gated chloride ion channels thus inhibiting GABA-induced chloride currents [11]. *In vitro* studies indicate that the isoxazoline binding site on arthropod GABA receptors is distinct from that of fipronil and so cross-resistance with other insecticides targeting GABA receptors is unlikely [11, 12]. The isoxazoline fluralaner was originally commercialized as an oral formulation for dogs to control fleas and ticks for 12 weeks following a single treatment [13, 14]. This extended activity of fluralaner *versus* monthly flea and tick products is believed to be a factor that can facilitate owner compliance with veterinary parasite control recommendations [15].

In recognition of the popularity of topically applied products among cat owners, a spot-on formulation of fluralaner was developed to treat and control flea and tick infestations in cats. The demonstrated efficacy of this formulation has been shown to parallel that which has been established for the chewable tablet formulation in dogs [6, 16, 17]. To further facilitate owner compliance with parasite control recommendations for cats, the most recent evolution in this product family has involved combining fluralaner (28% w/v) with an established endectocide, moxidectin (1.4% w/v), to prevent heartworm disease and to treat infections with intestinal roundworm and hookworm. As part of the development programme, a laboratory study was initiated to confirm the efficacy of this novel combination against a flea strain that had been recently isolated from a home in which there had been reported failure of owner-administered treatments with a combination product of fipronil and (*S*)-methoprene and imidacloprid. The objective of this study was to determine the immediate knockdown and persistent efficacy of fluralaner in cats against this flea isolate for 13 weeks following a single topical application of a combination spot-on of fluralaner and moxidectin.

## Methods

### Study design

This was a parallel group, randomized block, positive-controlled, non-blinded study conducted in accordance with Good Clinical Practice (VICH guideline GL9, Good Clinical Practice, EMA, 2000) guidelines [18]. The protocol was reviewed and approved by the study site Institutional Animal Care and Use Committee.

### Cats and housing

Thirty-two cats were screened for inclusion. All but one cat, which had been temporarily resident as a pet in the home of an investigator until being returned to the facility on Day −7, had been resident at the research facility for at least 3 months prior to the study. Each cat was microchipped, could be recognized individually by facility staff, and was assigned a unique identification number. The cats received a single nitenpyram treatment 12 days prior to enrolment to eradicate any environmental flea infestation they may have carried. There had been no other insecticidal treatments administered to the cats within the 60 days prior to enrolment. None of the cats had been treated at any time with an isoxazoline. Cats were required to be at least 6 months of age and to weigh at least 2 kg, to be in good health and good body condition, neither pregnant nor lactating, and to have shown flea-carrying capacity, as demonstrated in flea counts from the infestation used for randomization.

The cats had been maintained in groups in 4 exercise pens. Following allocation the cats were placed into individual cages. During the study compatible cats were grouped and placed in the exercise pens for up to 7 hours each day, with no contact possible between cats in different treatment groups. Cats were retained in their allocated cages during periods between flea infestations and counts. Food and water were provided in colour coded stainless steel bowls, and bowls remained with cats in the same treatment group throughout the study.

### Flea challenge

The Scarborough strain of *Ctenocephalides felis*, a field strain collected in 2015 from a household in Brisbane (Queensland, Australia) in which fipronil and (*S*)-methoprene and imidacloprid treatments had failed to control infestations on treated cats, was used as the challenge strain in this study. Prior to the study an *in vitro* bioassay had indicated that this flea strain may have been resistant to fipronil. There was no suggestion of any resistance to imidacloprid. The results were similar in 3 other bioassays conducted with this flea strain in a period of approximately 2 years after this study had concluded. In these bioassays the lethal concentrations that would kill 50% (LC_50_) and 90% (LC_90_) of fleas could be determined for permethrin (<5 ppm) and imidacloprid (<1 ppm), but could not be determined for fipronil as none of the tested concentrations (up to 3000 ppm) were able to kill more than 50% of the fleas. The cats were challenged with approximately 100 unfed adult *C. felis* on Days −6 (for selection and randomization), −1, 7, 14, 28, 42, 56, 70, 77, 84 and 91.

For flea counting, cats were sedated with a combination of tiletamine and zolazepam (Zoletil^®^, Virbac) to allow full-body comb counts to be completed 48 h after all but the Day −1 infestations when counts were completed 48 h post-treatment (i.e. 72 h post-infestation). Each cat was combed by 2 operators for at least 10 min, after which the counting procedure would be extended whenever either operator found a flea during the previous full minute of combing.

### Randomization and treatment

For randomization 32 cats were infested on Day −6 with approximately 100 fleas and ranked in descending order of counts that were completed on Day −4. The 2 cats with the lowest counts were excluded, and the remaining 30 were ranked by coat length and then by flea count, and formed into 10 blocks, each consisting of 3 cats. Within blocks cats were randomly allocated to three groups of 10 cats each: Group A cats were negative controls and received no treatment; Group B cats received a single topical treatment, on Day 0, with a spot-on formulation containing fluralaner plus moxidectin, administered according to body weight on Day −1 (to achieve a dose rate of 40 mg fluralaner and 2 mg moxidectin per kg); Group C cats were treated topically with a combination formulation of fipronil and (*S*)-methoprene (Frontline^®^ Plus for Cats, Boehringer Ingelheim, 0.5 ml) according to the manufacturer’s instructions.

Treatment was applied to the dorsal midline at the junction of the top of the neck and the base of the skull, taking care to apply the product directly to the skin. After treatment each cat was gently restrained for one minute. Cats were observed for 5 min after treatment for any behaviors that would indicate an adverse reaction to treatment or could affect efficacy due to loss of treatment such as rolling, shaking or rubbing. Each cat was inspected for run-off or streaking at approximately 30 min post-treatment (product running some distance from the application site, or hair coat wetness away from the application site). Safety assessments consisted of general health observations performed by trained staff at approximately 10-min intervals during the first hour after treatment, veterinary observations by a veterinarian at 2, 4 and 24 h post-treatment, and daily health observations at least twice a day by trained staff or a veterinarian as a part of routine maintenance at the cattery.

In each treatment group there were 2 domestic long-haired and 8 domestic short-haired cats, while the control group contained 1 domestic long-haired, 8 short-haired and 1 Himalayan cross cats. The age range in the fluralaner plus moxidectin group was 3.0–5.0 years, in the fipronil and (*S*)-methoprene group 2.5–5.9 years, and in the control group 2.8–5.4 years. Cat weights in each group were similar, ranging between 3.5–5.9 kg.

### Statistical assessments

The individual cat was the experimental unit. Efficacy was calculated by comparing flea counts at every post-treatment assessment of each treated group to the control group. Arithmetic and geometric means were calculated using the formula:

$${\text{Efficacy }}\left( \% \right) = 100 \, \times \left( {{\text{M}}_{\text{C}} - {\text{M}}_{\text{T}} } \right)/{\text{M}}_{\text{C}}$$

where M_C_ is the mean number of total adult live fleas on untreated cats, and M_T_ is the mean number of total adult live fleas on treated cats. Mean flea counts of the treated groups were also compared.

The flea count data were transformed prior to analysis using the Y = log_e_(x + 1) transformation. The log-transformed data were analyzed using a linear mixed model including treatment as the fixed effect and block as the random effect. Least squares means were used for treatment comparisons and were back transformed to obtain the estimates of geometric mean flea counts. A Kenward-Roger’s approximation was used to determine the denominator degree of freedom for the hypothesis. A two-tailed test was used for the comparison between treatment groups. Statistical significance was declared when the *P*-value was ≤ 0.05. The primary software used was SAS^®^ version 9.3 (SAS Institute Inc., Cary, NC, USA).

## Results

Fleas were found on all control group cats and all fipronil and (*S*)-methoprene group cats at every assessment. In the fluralaner plus moxidectin group, every cat was free of fleas throughout the post-treatment period, with the exception of Day 58 when 3 live fleas were found on a single cat. At all post-treatment assessments, fipronil and (*S*)-methoprene group individual flea counts ranged between 1–79 and control group counts ranged between 34–109 (Table 1).

**Table 1:** Summary of flea count data from cats in an untreated control group, a group treated topically on Days 0, 28 and 56 with fipronil and (*S*)-methoprene and a group treated topically on Day 0 with fluralaner plus moxidectin

Study day	Control			Fipronil/(S)-methoprene			Fluralaner plus moxidectin		
	Arithmetic mean ± SD	Geometric mean	Range	Arithmetic mean ± SD	Geometric mean	Range	Arithmetic mean ± SD	Geometric mean	Range
Pre-Tx	56.2 ± 10.7	55.3	44–76	54.9 ± 10.8	54.0	42–74	54.8 ± 9.9	54.1	43–76
2	70.0 ± 14.0	68.6	42–93	43.4 ± 25.0	35.2	7–79	0 ± 0	0	na
9	74.4 ± 12.7	73.4	55–94	34.5 ±19.3	27.5	3–61	0 ± 0	0	na
16	79.4 ± 15.3	78.0	51–109	31.1 ± 14.0	26.8	4–56	0 ± 0	0	na
30	67.4 ± 15.0	66.0	49–92	34.1 ± 16.0	26.7	1–55	0 ± 0	0	na
44	68.6 ± 14.6	67.1	40–95	28.1 ± 11.4	24.2	3–39	0 ± 0	0	na
58	56.1 ± 15.3	54.5	40–92	31.5 ± 9.9	30.3	21–54	0.3 ± 0.9	0.1	0–3
72	66.1 ± 21.1	63.2	38–100	28.0 ± 11.9	25.0	6–42	0 ± 0	0	na
79	63.2 ± 19.1	60.3	34–89	34.0 ± 12.5	31.3	9–55	0 ± 0	0	na
86	70.3 ± 17.0	68.3	41–90	41.3 ± 18.0	35.0	5–64	0 ± 0	0	na
93	63.6 ± 21.6	60.5	39–92	46.6 ± 17.8	42.0	9–64	0 ± 0	0	na

*Abbreviations*: na, not applicable; SD, standard deviation

**Fig. 1:**
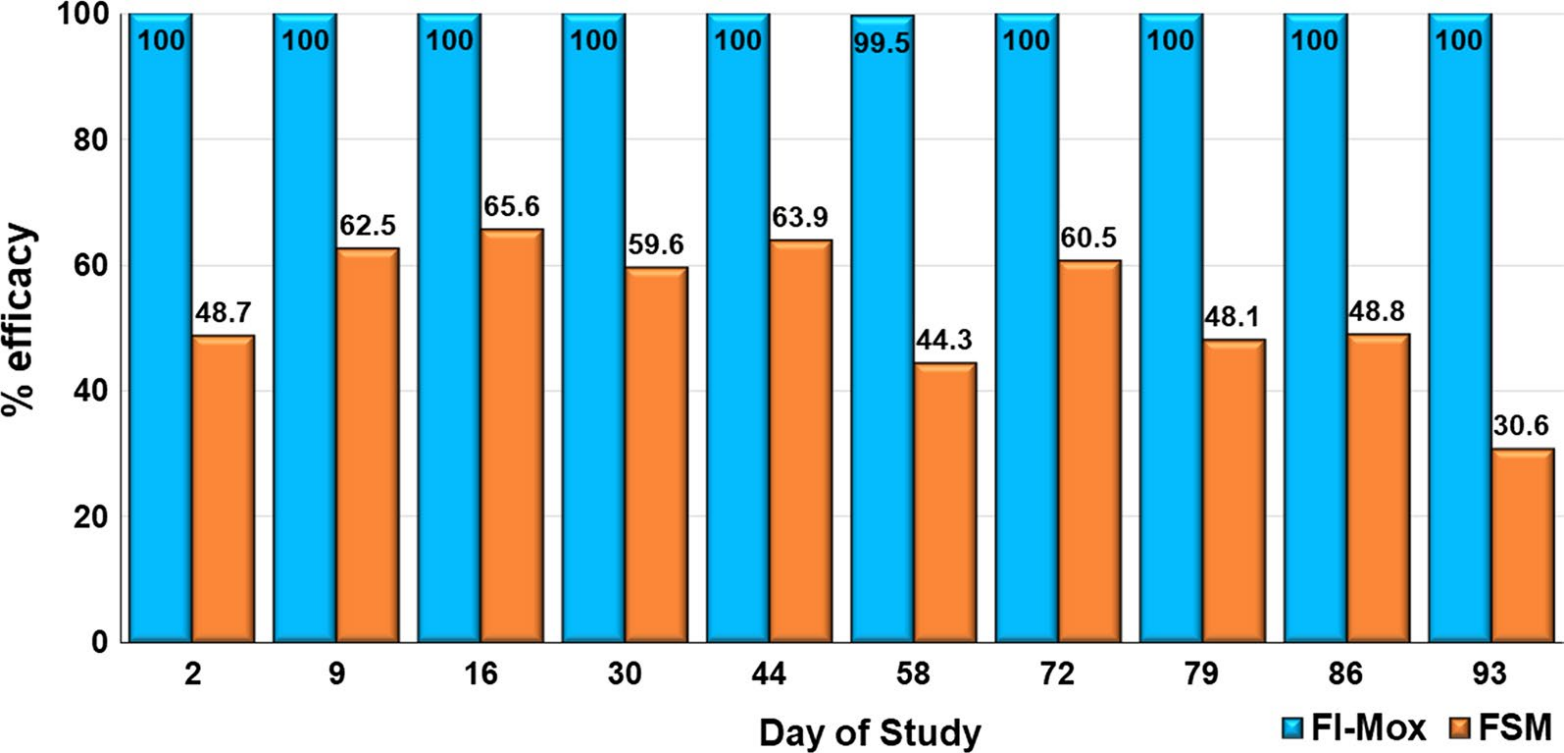
Percent efficacy based on geometric mean *Ctenocephalides felis* counts relative to an untreated control group at 48 h after treatment and weekly post-treatment re-infestation for cats treated with either a single topical dose of fluralaner  plus moxidectin on Day 0 or three treatments with topical fipronil and (*S*)-methoprene on Days 0, 28 and 56

At each post-treatment assessment, compared to the control group, the differences in mean flea counts were significantly lower in both the fluralaner plus moxidectin group (*P* < 0.001) and the fipronil and (*S*)-methoprene group (*P* ≤ 0.04) (Table 2). At all assessments, the mean flea counts in the fluralaner plus moxidectin group were significantly less than those of the fipronil and (*S*)-methoprene group (*P* < 0.001). The efficacy of fluralaner plus moxidectin was 100% at all post treatment assessments on every occasion except on Day 58 when geometric mean efficacy was 99.7% (arithmetic mean efficacy 99.5%). The geometric mean efficacy of fipronil and (*S*)-methoprene ranged between 30.6–65.6% (arithmetic mean efficacy 26.7–60.8%) (Table 2, Fig. 1).

**Table 2:** Between-group comparison of geometric mean flea counts from cats in an untreated control group, a group treated topically on Days 0, 28 and 56 with fipronil and (*S*)-methoprene, and a group treated topically on Day 0 with fluralaner plus moxidectin

Study day	Fipronil and (*S*)-methoprene *vs* control				Fluralaner plus moxidectin *vs* control				Fluralaner plus moxidectin *vs* fipronil and (*S*)-methoprene	
	*t*-value	*P*-value	% Efficacy		*t*-value	*P*-value	% Efficacy		*t*-value	*P*-value
			GM	AM			GM	AM		
2	*t*_(27)_ = 3.3	0.003	48.7	38.0	*t*_(27)_ = 21.1	<0.001	100	100	*t*_(27)_ = −17.9	<0.001
9	*t*_(18)_ = 4.6	<0.001	62.5	53.6	*t*_(18)_ = 20.7	<0.001	100	100	*t*_(18)_ = −16.1	<0.001
16	*t*_(18)_ = 6.5	<0.001	65.6	60.8	*t*_(18)_ = 27.2	<0.001	100	100	*t*_(18)_ = −20.7	<0.001
30	*t*_(18)_ = 3.5	0.003	59.6	49.4	*t*_(18)_ = 16.6	<0.001	100	100	*t*_(18)_ = −13.1	<0.001
44	*t*_(18)_ = 5.3	<0.001	63.9	59.0	*t*_(18)_ = 22.4	<0.001	100	100	*t*_(18)_ = −17.1	<0.001
58	*t*_(18)_ = 4.2	<0.001	44.3	43.9	*t*_(18)_ = 28.8	<0.001	99.7	99.5	*t*_(18)_ = −24.5	<0.001
72	*t*_(27)_ = 5.5	<0.001	60.5	57.6	*t*_(27)_ = 25.2	<0.001	100	100	*t*_(27)_ = −19.8	<0.001
79	*t*_(18)_ = 4.7	<0.001	48.1	46.2	*t*_(18)_ = 30.2	<0.001	100	100	*t*_(18)_ = −25.5	<0.001
86	*t*_(27)_ = 3.3	<0.001	48.8	41.3	*t*_(27)_ = 21.2	<0.001	100	100	*t*_(27)_ = −17.9	<0.001
93	*t*_(27)_ = 2.1	0.041	30.6	26.7	*t*_(27)_ = 24.7	<0.001	100	100	*t*_(27)_ = −22.5	<0.001

*Abbreviations*: GM, geometric mean; AM, arithmetic mean

There were 10 adverse events recorded in all groups during the study, none of which were deemed treatment-related. Mostly the adverse events (*n* = 7) were described as patchy hair loss caused by flea irritation and/or combing. The remaining 3 included a pyometra, cat flu and a vulval tear. No product was observed streaking, running or dripping off following topical application that would be suggestive of loss of any of the applied dose.

## Discussion

Fluralaner plus moxidectin provided 100% efficacy (99.7% at 58 days) against repeated flea challenges for 13 weeks following a single topical application. These results provide further evidence of the high and consistent long duration of efficacy of topically applied fluralaner in the treatment and control of flea infestations in cats. The results are consistent with the findings of two field studies in client-owned cats in the USA in which the field performance of the topical formulation containing only fluralaner has been compared to other topically applied parasiticides. In one study where treatment was applied by cat owners, a single owner-application of fluralaner spot-on provided at least a 99.0% reduction in geometric mean flea counts from two to 12 weeks post-treatment [6]. This was compared to three consecutive monthly treatments with a combination of fipronil and (*S*)-methoprene that provided a reduction in geometric mean flea counts between 55.2–75.4% [6]. At the end of that study, 80% of cats in the fluralaner group were free of fleas compared to just 23.5% of cats in the fipronil and (*S*)-methoprene group. In the second study, the efficacy of a single topical application of fluralaner against fleas was compared to that provided by three consecutive monthly applications of selamectin [17]. In the fluralaner group, geometric mean flea counts were reduced by 96.6% at 7 days, and by 100% at approximately 12 weeks post-treatment. This was compared to reductions in flea counts of 79.4% within 7 days and 91.3% following three consecutive monthly applications of selamectin [17].

The results that we report are particularly relevant because they demonstrate fluralaner efficacy against a flea isolate that, under field conditions, was not controlled by repeated treatments with fipronil and (*S*)-methoprene. This decline in fipronil efficacy has been noted in other field studies. In the present study, all of the cats treated with fipronil had at least 1 and up to 79 fleas, and efficacy (based on geometric mean flea counts) was 59.6, 44.3 and 48.8% at 48 hours following the treatments at 4, 8 and 12 weeks, respectively. There is one other report in which a field-collected flea isolate from a suspected fipronil failure case was subjected to a laboratory challenge [2]. In that study the efficacy of fipronil spray and spot-on formulations, based on flea counts at 48 hours following infestation, was high in the two weeks following treatment, but declined to less than 95% at 21 days post-treatment. At 28 days post-treatment efficacy was 29.7 and 48% for the spray and spot-on formulations, respectively. Those findings of a decline in fipronil efficacy later in the month following treatment align with another laboratory study that tested the efficacy of a fipronil spray against the KS1 flea strain, a laboratory-maintained isolate that had been collected six years prior to the availability of fipronil for use in pets [19].

In the present study care was taken to ensure that products were applied strictly in accordance with label recommendations, with close post-treatment monitoring to verify that there was no loss of product. There was no contact between cats in different groups, and there was no opportunity for infestation with fleas from external sources. The results therefore remove the role of incorrect product application as a cause of fipronil failure against this flea isolate and add to the literature reporting the failure of fipronil to adequately control fleas. Importantly in the study we present, the fleas that were not controlled by fipronil and (*S*)-methoprene were fully susceptible to fluralaner plus moxidectin.

## Conclusions

Treatment with fluralaner plus moxidectin provided 99.7 to 100% efficacy against fleas for 13 weeks following a single application. These findings demonstrate complete fluralaner plus moxidectin efficacy against a flea isolate that was not controlled by fipronil and (*S*)-methoprene either in field use or under these study conditions. This study provides further confirmation of the consistent and sustained efficacy of topically applied fluralaner in the treatment and control of flea infestations in cats.

## Data Availability

Data from this study are proprietary and maintained by Merck Animal Health, Madison, NJ, USA.

## Abbreviations

VICH: Veterinary International Cooperation on Harmonisation
EMA: European Medicines Agency
GABA: γ-aminobutyric acid
GM: geometric mean
AM: arithmetic mean
LC: lethal concentration
M_C_: mean number of live fleas on untreated cats
M_T_: mean number of live fleas on treated cats
SD: standard deviation
